# Gut phages are linked to off-target antimicrobial resistance genes in orthopedic patients receiving peri-surgical antibiotics

**DOI:** 10.3389/fmicb.2026.1892653

**Published:** 2026-09-09

**Authors:** Afroditi Kouraki, Christopher Deacon, Jessica Nightingale, Panayiotis Louca, Tony Kelly, Amy Zheng, Alexia Karantana, Noor Kifah Al-Tameemi, Amrita Vijay, Aitor Blanco-Míguez, Davide Bazzani, Mattia Bolzan, Angela Bonadiman, Guendalina Tonidandel, Teresa Vanzo, Suzanne Miller, Benjamin Ollivere, Cristina Menni, Ana M. Valdes

**Affiliations:** 1 NIHR Nottingham Biomedical Research Centre, Queens Medical Centre, Nottingham, United Kingdom; 2 Injury, Recovery and Inflammation Sciences, School of Medicine, University of Nottingham, Nottingham, United Kingdom; 3 Department of Twin Research, Ageing and Genomic Epidemiology, King’s College London, London, United Kingdom; 4 Trauma and Orthopaedics, Nottingham University Hospitals, Nottingham, United Kingdom; 5 Prebiomics S.r.l., Trento, Italy; 6 Department of Pathophysiology and Transplantation, Università Degli Studi di Milano, Milan, Italy; 7 Fondazione IRCCS Cà Granda Ospedale Maggiore Policlinico, Angelo Bianchi Bonomi Hemophilia and Thrombosis Center, Milan, Italy

**Keywords:** antibiotic resistance genes, antibiotics, antimicrobial resistance, genomics, microbiome, phages, trauma, viral sequence groups

## Abstract

**Introduction:**

Antibiotic prophylaxis alters the gut microbiome, potentially expanding antimicrobial resistance genes (ARGs). The role of bacteriophages in this process remains poorly understood. This study investigates the effects of predominantly flucloxacillin- and gentamicin-based perioperative antibiotic prophylaxis on phage and ARG dynamics in orthopedic trauma patients.

**Methods:**

Shotgun metagenomic analysis in two orthopedic cohorts: rib fracture fixation (*n* = 16) and frail hip fracture fixation (*n* = 22), versus frail (*n* = 18) and non-frail (*n* = 95) controls was performed. Phage abundance (viral sequence groups [VSGs] relative abundance) was quantified using MetaPhlAn 4.1, while ARG abundance using ResFinder 7 (v2022) with KMA (v2.3.0). ViroProfiler with CheckV for quality assessment was used to detect ARG-carrying viral contigs in antibiotic-treated patients.

**Results:**

Following antibiotics, seven VSGs were increased in rib and five in the frail hip fractures compared to controls (mean Log₂ Fold change: 16.89 and 17.21, respectively). In the frail cohort, 18 ARGs increased, including 14 off-target ARGs (resistance genes for antibiotics not administered; mean Log₂ Fold change >25 compared to controls). Five VSGs correlated with six off-target ARGs in antibiotic-treated patients (Rho = 0.612). Longitudinal analysis of rib fracture patients (*n* = 10) revealed off-target ARGs increased from discharge to follow-up, paralleling increased VSGs abundances, while on-target ARGs remained mostly stable. In antibiotic-treated compared to controls, phage-related and DNA-repair gene families were enriched, phage gene richness was associated with stress and biofilm formation pathways, and VSGs correlated with opportunistic species (e.g., *E. coli*). More frail antibiotic-exposed patients (81.5%) carried ARG-containing viral contigs than non-frail patients (41.4%).

**Discussion:**

Our findings suggest perioperative antibiotics may contribute to phage-associated dissemination of off-target ARGs. Understanding how ARGs spread in the gut microbiome is important for improving antibiotic stewardship and protecting vulnerable patients from difficult to treat infections.

## 1 Introduction

In 2019, antimicrobial resistance (AMR) was directly responsible for an estimated 1.27 million deaths worldwide and projections suggest it could rise to 10 million annually by 2050. AMR reduces the efficacy of standard treatments, therefore persistent infections raise the risk of disease spread, severe illness, and death (Willems et al., 2023). Both commensal and pathogenic bacteria can carry AMR genes (ARGs), which can spread to other microorganisms (Penders et al., 2013).

Oral antibiotic treatment in healthy individuals without active infection increases the prevalence of ARGs, affecting both “on-target” (class-related) and “off-target” (unrelated) genes, and transiently reduces gut microbial diversity, with recovery usually within a few months (Anthony et al., 2022). However, in major musculoskeletal injuries which require surgical fixation, antibiotic therapy induces persistent gut microbiome dysbiosis and a sustained increase in acquired ARGs lasting more than 12 weeks. Importantly, while some “on-target” ARGs were limited to a handful of species, many off-target ARGs are detected across a wide range of commensal and pathobiont species (Kouraki et al., 2024).

Bacteriophages (phages) regulate gut microbiome dynamics (Kirk et al., 2024) and contribute to horizontal gene transfer. DNA-damaging antibiotics, like fluoroquinolones, can induce prophages to trigger a switch from lysogeny to lysis. In addition to DNA damage responses, many intestinal phages respond to different environmental cues, such as temperature, pH levels, reactive oxygen species and inflammation (Zhang et al., 2000). ARGs are often encoded within phage genomes, suggesting phages may contribute to ARG dissemination through both horizontal and vertical gene transfer (Liao et al., 2024). Recent evidence using nanopore sequencing demonstrates that capsid-mediated packaging of bacterial DNA is widespread in the gut microbiome, with up to 5.4% of virome DNA being of bacterial origin (Borodovich et al., 2026).

Despite these links, the effects of antibiotics on prophage induction and horizontal ARG transfer remain poorly defined, and the specific bacterial targets and pathways involved are not fully understood (Münch et al., 2023; Modi et al., 2013; Maurice et al., 2013). Recent work by Heston et al. (2024) proposed that antibiotics directed mainly at aerobic bacteria are less likely to promote off-target antimicrobial resistance than agents with anaerobic coverage.

**Figure 1:**
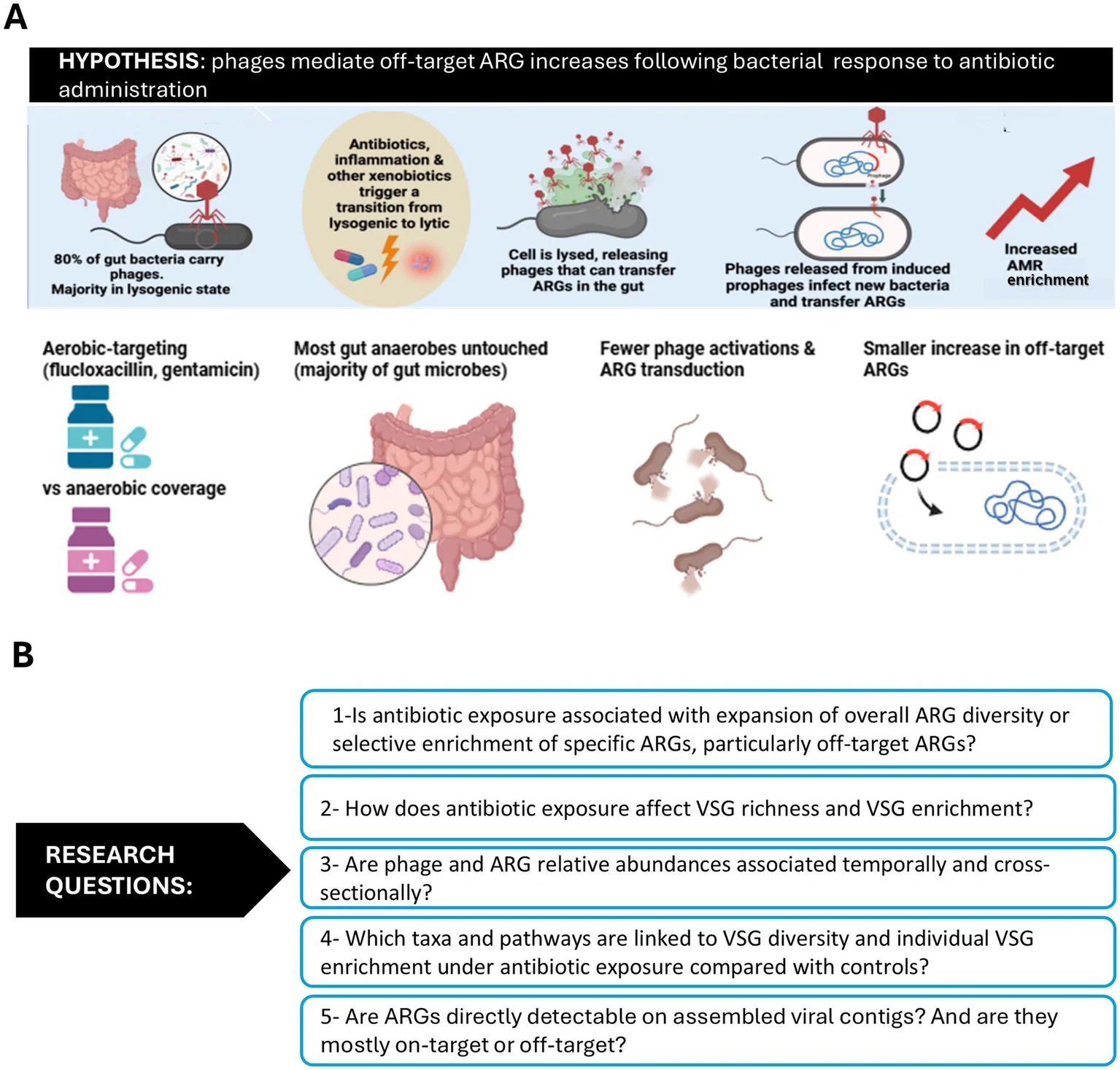
Study hypothesis and conceptual model. **(A)** Antibiotic administration may trigger bacterial stress responses that induce prophages, leading to increased phage release. These phages may carry antimicrobial resistance genes (ARGs), potentially contributing to the expansion of both on-target and off-target resistance. At the same time, it has been proposed that antibiotics with limited anaerobic coverage may be less likely to trigger off-target antimicrobial resistance through phage-mediated ARG transfer. The prophylactic regimen studied here (mainly flucloxacillin + gentamicin) predominantly targets aerobic bacteria, offering an opportunity to test this prediction in a clinical setting. **(B)** Research questions based on the hypotheses.

We hypothesize that prophylactic antibiotic administration may promote bacterial stress responses, and in turn, phage activation (most likely lysogenic), which may contribute to increased phage diversity and enrichment and a potential role for phage-mediated horizontal transfer of ARGs (Figures 1A,B). To test this hypothesis, we examined whether: (i) antibiotic exposure is associated with changes in both phage and ARG diversity and relative abundance, (ii) phage diversity and relative abundance is associated with ARG diversity and relative abundance, including longitudinal changes in off-target ARGs following antibiotic exposure, (iii) specific phages are more strongly associated with off-target ARGs (resistance to non-administered antibiotics) than on-target ARGs, (iv) ARGs can be detectable on assembled viral contigs, providing physical evidence supporting phage-ARG linkage.

In this study, orthopedic trauma patients receiving predominantly flucloxacillin- and gentamicin-based perioperative prophylaxis were compared to matched controls to test these research questions. Analyses focused on differences in on- and off-target ARG relative abundances, their links to specific viral sequence groups (VSGs), the relationship between antibiotic use and phage-related gene families, DNA repair and SOS response gene families, as well as bacterial metabolic pathways and species associated with VSG richness or specific VSGs under antibiotic exposure. Data were drawn from two orthopedic cohorts receiving pre-surgical systemic antibiotic administration and two control datasets (of non-injured healthy and age-matched individuals).

## 2 Methods

### 2.1 Study cohorts

#### 2.1.1 Patients with multiple rib fractures (OPERA cohort)

**Figure 2:**
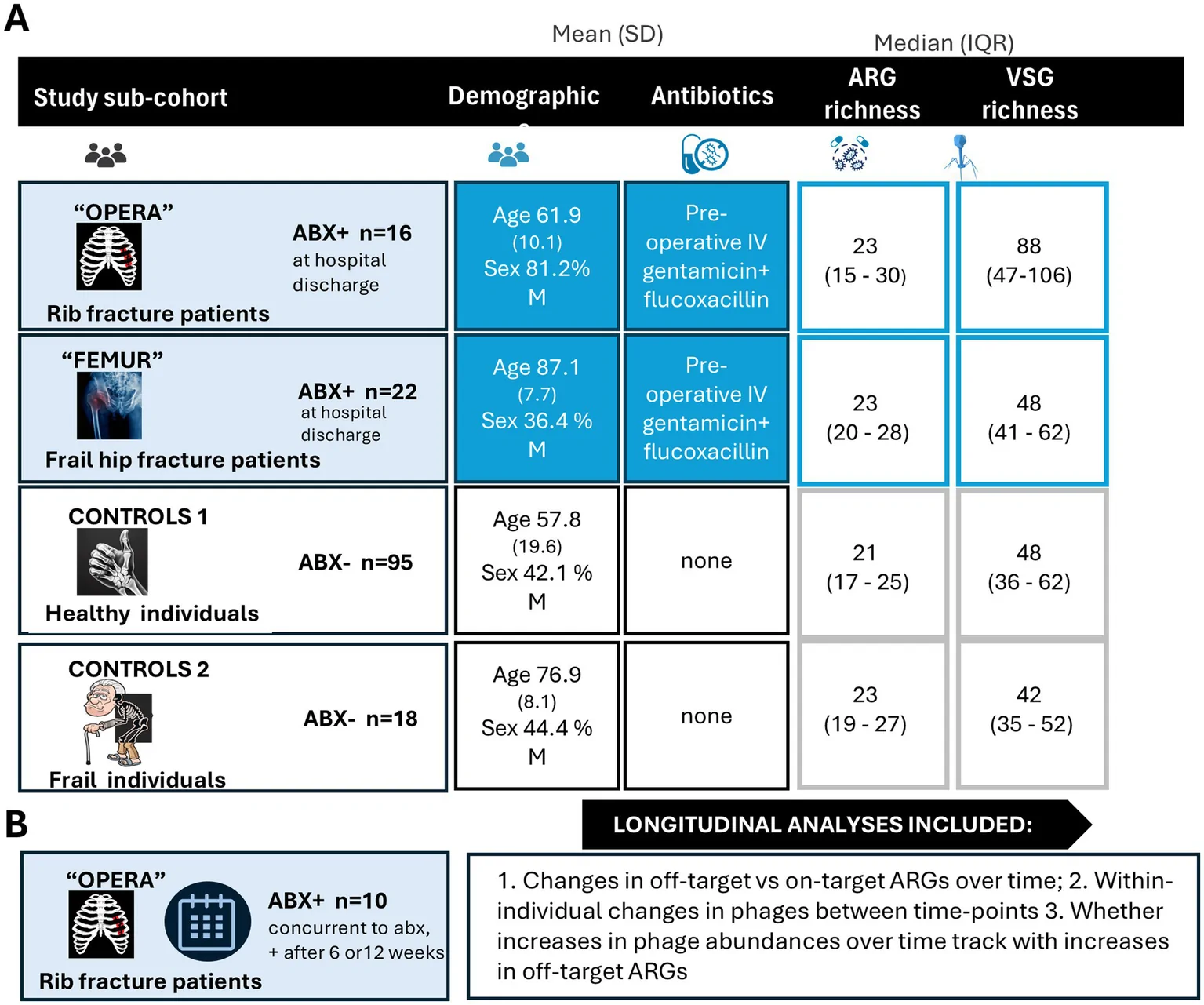
Study cohorts and longitudinal design. **(A)** Study cohorts: rib fracture patients receiving perioperative antibiotics (“OPERA,” *n* = 16), frail hip fracture patients receiving antibiotics (“FEMUR,” *n* = 22), and two antibiotic-free control groups (healthy controls, *n* 95; frail controls, *n* = 18). Key demographic features (mean, SD), antibiotic exposure, and summary measures (median, IQR) of antimicrobial resistance gene diversity and viral sequence group gene richness are shown **(B)** Longitudinal analyses: a subset of rib fracture patients who received antibiotics (*n* = 10) were followed from immediately after antibiotic exposure (hospital discharge) to 6 and/or 12 weeks later. These data allowed us to examine: (1) Whether off-target ARGs increased more strongly than on-target ARGs over time, (2) Within-individual changes in VSG richness after antibiotics, and (3) Whether increases in the abundances of specific VSGs were linked to subsequent increases in ARGs. ABX, antibiotics; ARG, antimicrobial resistance genes. VSG, viral sequence group.

This cohort has been described in detail previously (Kouraki et al., 2024; Ollivere, 2019). Briefly, it included adults with ≥3 rib fractures, as defined by British Orthopedic Association Standards for Trauma (British Orthopaedic Association, 2015), recruited from a UK Level 1 Trauma Center as part of *The Operative Rib Fixation* (OPERA) study, a trial comparing non-operative management with early surgical rib fixation. Stool samples from 21 participants were analyzed at discharge, 6 weeks, and 12 weeks post-discharge. Of these *n* = 16 had received prophylactic antibiotics during their hospital stay and were used in the current analysis. In addition, 10 participants with longitudinal samples collected immediately after antibiotic exposure (hospital discharge) and again at 6 or 12 weeks (five with all three timepoints) were used to assess temporal links (Figure 2).

#### 2.1.2 Frail hip fractures (FEMUR cohort)

The frail hip fracture cohort comprises 22 patients recruited from the *Functioning of Elder Muscle; Understanding Recover*y (FEMUR) study (Ollivere, 2025). The study enrolled frail older adults admitted with an operatively managed hip fracture. All participants were >65 years old, with a clinical frailty score of ≥4. All patients were recruited from Nottingham University Hospitals National Health Service (NHS) Trust. For the present analysis, all participants had provided stool samples and had received prophylactic antibiotics during their hospital stay (see Figure 2A for descriptive characteristics).

#### 2.1.3 Frail controls

Eighteen frail or pre-frail participants from the *Link between Frailty and Gut Microbiome in Older Adults* (FLORA) (*n* = 11) and from the *Effect of Pectin Supplementation on Geriatric with Frailty: A Randomized Placebo-Controlled Dietary Intervention* (GEOFF) study (Valdes, 2025a) (*n* = 7). All participants were over 60 years old and had previously completed the FRAIL questionnaire as part of a larger survey. Criterion sampling was used to select frail/pre-frail participants. Current frailty status assessed using the FRAIL scale was reconfirmed and used to classify the 18 individuals who provided stool samples for the current analysis (please refer to Supplementary Table S1 for FLORA’s recruitment criteria).

#### 2.1.4 Healthy controls

Samples from 40 community-dwelling individuals in the INSPIRE study (Valdes, 2025b) (two timepoints; *n* = 80) and 41 individuals from two pectin dietary intervention studies (Vijay et al., 2024) (two timepoints; *n* = 81). In addition, samples from 14 robust non-frail individuals from the FLORA study that were age-matched to the frail/prefrail group.

### 2.2 Ethical considerations

All studies were conducted in accordance with the Declaration of Helsinki. The OPERA ORiF [REC ref. 18/SC/066, IRAS 248460, ISRCTN 10777575 (Ollivere, 2019)] and Femur II [REC ref. 20/LO/0841, IRAS 268635, NCT04764617 (Ollivere, 2025)] were approved by NHS Health Research Authority (HRA) and REC. The control cohorts [FLORA REC ref. FMHS 230–0624; INSPIRE REC ref. 475-0322, NCT05670314 (Valdes, 2025b); GEOFF REC ref. FMHS 280-0924, NCT06955975 (Valdes, 2025a); PECTIN studies REC ref. FMHS 302-0621/FMHS 287-0523 (Vijay et al., 2024)] were approved by the Institutional Ethics Committee at University of Nottingham Faculty of Medicine and Health Sciences Research Ethics Committee. All participants provided written informed consent.

### 2.3 Antibiotic use

Antibiotic use for the patients was recorded as part of the study. In total, 38 antibiotic-treated individuals from the OPERA and FEMUR cohorts and 113 antibiotic-free individuals from the FLORA, PECTIN, INSPIRE, GEOFF studies were used in analysis. The specific antibiotics administered and duration are listed in Supplementary Table S2. Control participants completed detailed medical questionnaires documenting recent medication use, with none having received antibiotic treatment within 3 months prior to sampling.

### 2.4 Stool sample collection

Stool samples were collected from orthopedic surgery patients at discharge (and at 6- and 12-week follow-up for rib fracture patients) and from control individuals using standardized collection kits and protocols. Participants were provided with stool collection kits and detailed instructions for sample collection and postal return. All samples were stored at −80 °C according to local standard operating procedures at the Clinical Science Building (City Hospital, Nottingham). Samples were then shipped to Prebiomics (Trento, Italy) for DNA extraction, sequencing quality control, preprocessing, and bioinformatic analyses.

### 2.5 DNA extraction, library preparation and sequencing

Microbial DNA was extracted from stool samples using the DNeasy 96 PowerSoil Pro QIAcube HT kit (Qiagen, #47021). DNA concentration was quantified using the Quant-iT dsDNA Assay Kit (Life Technologies) on a Varioskan LUX microplate reader (Thermo Fisher Scientific, #VL0000D0). Sequencing libraries were prepared using the Illumina DNA Prep DNA Prep, (M) Tagmentation (96 Samples, IPB) kit (Illumina, #20060059) followed by double-sided bead purification according to the manufacturer’s protocol. The Quant-iT 1X dsDNA Assay Kits, HS (Life Technologies, #Q33232) and the Varioskan LUX Microplate Reader (Thermo Fisher Scientific, #VL0000D0) were used to determine the DNA concentration of these libraries. In addition, base pair length was determined using the D5000 ScreenTape Assay (Agilent, #5067–5,588/9) in conjunction with the TapeStation 4,150 (Agilent Technologies, #G2992AA). Libraries were pooled using the Qubit 1x dsDNA HS kit (Life Technologies, #Q33231) with the Qubit 3.0 Fluorometer (Life Technologies, #Q33216) and sequenced as paired-end reads on the Illumina NovaSeq 6000Dx platform, targeting an average depth of ~15 Gb/sample.

### 2.6 Preprocessing and host DNA removal

Preprocessing of sequencing reads was performed using standardized pipelines accessible at https://github.com/SegataLab/preprocessing. In particular, raw sequencing reads underwent read level quality control and adapter trimming using TrimGalore (v0.6.6) (Krueger et al., 2023). Reads were removed if they:

- Had a quality score <20
- Were shorter than 75 bp after trimming
- Contained >5 ambiguous nucleotides

To remove host contamination, filtered reads were aligned to the human reference genome (hg19) using Bowtie2 (v2.3.4.3) (Langmead and Salzberg, 2012) with default parameters. Reads mapping to the human genome or to Illumina PhiX spike-in sequences were discarded. Cleaned reads were subsequently split and sorted.

### 2.7 Taxonomic profiling

Microbial taxonomic composition was assessed using the MetaPhlAn 4.1 software tool with the mpa_vOct22 marker database (Blanco-Míguez et al., 2023), which quantifies microbial taxa based on clade-specific marker genes representing species-level genome bins (SGBs). The mpa_vOct22 database comprises dereplicated genomes (including metagenome-assembled and reference genomes) clustered into family-, genus-, and SGBs. To minimize artifacts, unknown SGBs were retained only when supported by at least five metagenome assembled genomes.

In the construction of the MetaPhlAn marker database by its developers, genomes in the catalogue were annotated using the UniRef90 database, and genes not assigned to existing UniRef90 families were de-novo clustered using UniClust90 (>90% identity and >80% coverage). Core genes present in the majority of genomes within each SGB were identified and screened against the entire genome catalogue to define SGB-specific marker genes, resulting in a set of approximately 5.1 million clade-specific markers used for taxonomic profiling. Metagenomic reads were mapped to these markers using Bowtie2 (Langmead and Salzberg, 2012) to estimate relative abundances of microbial taxa.

### 2.8 Functional metagenomic profiling

Functional potential was evaluated using HUMAnN (v3.8) (Beghini et al., 2021). Following the standard HUMAnN workflow, quality-filtered reads were first aligned at the nucleotide level to the ChocoPhlAn pangenome database (Blanco-Míguez et al., 2023). Reads not mapping to known species were then translated and aligned to the UniRef90 protein database using DIAMOND (Beghini et al., 2021), enabling quantification of microbial gene families as well as metabolic pathways using the MetaCyc pathway database (Karp et al., 2002). Gene family abundances were extracted and used as reads per kilobase. Functional annotations related to bacteriophage protein families and DNA repair/SOS damage response-associated gene pathways were filtered from the HUMAnN output tables (Beghini et al., 2021).

### 2.9 Antimicrobial resistance genes profiling

AMR genes across participants’ microbiomes and their prevalence in the microbial communities were profiled by aligning quality-controlled reads to the ResFinder 7 database (version 2022) (Clausen et al., 2018) using KMA (version 2.3.0) (Florensa et al., 2022), which enables identification of both acquired resistance genes and chromosomal mutations associated with antimicrobial resistance based on sequence similarity and provides species-specific resistance predictions for selected pathogens, ensuring that reported resistance determinants correspond to clinically relevant phenotypes.

To ensure robust detection, only matches meeting predefined thresholds were retained, including ≥90% sequence identity, sufficient alignment coverage, and a minimum template depth of ≥5-fold, consistent with thresholds previously used for KMA-based AMR gene detection (Clausen et al., 2018). Gene abundances were subsequently normalized using the reads per kilobase million metric to account for gene length and sequencing depth.

ARGs were classified as on-target when associated with resistance to an antibiotic class administered to the corresponding participant, and as off-target when they conferred resistance to antibiotic classes not administered. All recorded antibiotic exposures were considered in this classification (Supplementary Table S2).

### 2.10 Viral profiling

Viral profiling was performed using MetaPhlAn 4.1 with the “--profile_vsc” option, which maps reads to the curated VSG catalogue derived from assembled gut viromes (Zolfo et al., 2024), after preprocessing to remove low-quality reads. The MetaPhlAn 4.1 VSG reference catalogue, as described by Zolfo et al. (2024), was constructed from a large collection of viral-like particle (VLP)–enriched metagenomic samples. In total, more than 3,000 VLP-enriched viromes were evaluated to generate a comprehensive set of high-quality viral sequences included in the MetaPhlAn 4.1 database.

In the construction of the MetaPhlAn VSG catalogue by its developers, viromes were screened for viral enrichment using ViromeQC, and highly enriched samples (>50x) underwent metagenomic assembly. Specifically, 255 highly enriched viromes were assembled, producing 120,041 contigs, which were subsequently screened to remove residual non-viral sequences and clustered at 90% sequence identity. These clusters were further expanded using closely related sequences detected across 9,428 metagenomes and 2,789 additional viromes with lower enrichment. Representative sequences from these clusters were selected, resulting in a catalogue of 45,872 viral sequences grouped into 3,944 viral clusters. VSGs were then defined using a graph-based clustering approach based on sequence similarity.

Each VSG is denoted by a unique number following the letter “M.” These M number refer to internal identifiers assigned to each VSG in the MetaPhlAn reference database and do not correspond to established viral taxonomy or a specific phage classification scheme (Zolfo et al., 2024). The resulting catalogue captures a broad diversity of human gut bacteriophages, including both previously characterized phages (14.9% with RefSeq genomes) and a large fraction of previously uncharacterized viral sequences (85.1%).

#### 2.10.1 Database and reproducibility

All VSG definitions, sequence memberships, and associated metadata are publicly available through the MetaPhlAn database resources (Zolfo et al., 2024) and can be detected directly from metagenomes using the MetaPhlAn 4.1 --profile_vsc option.

To quantify viral and ARG diversity while reducing compositional bias inherent in relative abundance metrics, we calculated gene richness as the primary diversity measure. Gene richness represents the count of distinct genetic elements detected per sample above defined detection thresholds, providing a metric that is less sensitive to sequencing depth variation when samples are sequenced to comparable coverage (Finn, 2024).

#### 2.10.2 VSG gene richness

VSG richness was defined as the number of distinct VSGs/sample (Debédat et al., 2019; Zimmerman et al., 2023) and served as our measure of gut phage diversity.

#### 2.10.3 ARG richness

ARG richness was defined as the number of distinct ARGs/sample using KMA alignment against the ResFinder database and was used as a measure of resistome diversity (Debédat et al., 2019; Zimmerman et al., 2023).

### 2.11 Bacterial taxonomic alpha diversity

The Shannon diversity index was computed at the species level using the estimate_richness function from the phyloseq package (version 1.44.0) (McMurdie and Holmes, 2013) in R (version 4.4.1).

### 2.12 Alternative assembly pipeline to evaluate viral carriage of ARGs

Quality-controlled metagenomic reads from 38 antibiotic-exposed individuals were assembled individually (sample-level assemblies) with a 3 kb minimum contig length. Viral contigs were identified using ViroProfiler, quality assessed with CheckV to retain medium-to-high quality sequences and screened for ARGs using ResFinder 7 database [version 2022 (Florensa et al., 2022)] with ≥90% identity and ≥60% coverage thresholds. ARG-carrying contigs were defined as medium-to-high quality viral sequences containing at least one annotated resistance gene meeting these criteria.

### 2.13 Differential abundance of AMR genes and viral sequence groups

Differential abundance testing of ARGs and phages was performed to assess changes in ARG and VSG abundance after antibiotic treatment compared with controls, using ‘DESeq2’ (Differential Expression analysis for Sequencing data) package (version 1.40.2), with the “poscounts” normalization estimator to account for sparsity in metagenomic count data (Anders and Huber, 2010; Love et al., 2014). Analyses were adjusted for antibiotic-exposed versus antibiotic-free groups allowing us to test whether observed changes in ARGs and phages are attributable to antibiotic usage. For DESeq2, analyses were restricted to age- and sex-matched case–control samples processed within the same sequencing batch. Restricting analyses to matched samples within batch minimized imbalance between comparison groups and reduced potential confounding due to age, sex, frailty, and batch effects. Consequently, batch was not included as a covariate in the DESeq2 models.

### 2.14 Gene richness for ARGs and VSGs by antibiotic use

To assess the relationship between VSG richness and ARG richness we performed linear regression using gene richness estimates for VSGs as the predictor and ARG richness as the outcome. The model was adjusted for age, sex, visit number, Shannon alpha diversity, and batch effects. Shannon diversity was included as a covariate to account for overall microbial community diversity, and model assumptions were assessed by inspection of the residuals. To evaluate whether ARG richness and VSG gene richness were associated with antibiotic exposure status, we applied logistic regression models using antibiotic exposure as the outcome. ARG and VSG richness were tested separately as predictors in models adjusted for the same covariates as above. Model performance was evaluated using odds ratios and 95% CIs. These analyses included samples from 151 individuals (*n* = 38 antibiotic-treated and *n* = 113 antibiotic-free individuals).

### 2.15 Phage gene richness and bacterial pathways interactions

To investigate associations between bacterial pathways and VSG richness, we filtered pathways by a minimum mean relative abundance threshold of 0.1, which retained 30,811 pathways from 41,571. Mixed-effects linear models were applied using the *lmerTest* package in R. Models included fixed effects for VSG richness, antibiotic group, and their interaction, and were adjusted for batch effects with a random intercept for each subject. The Benjamini–Hochberg false discovery rate (FDR) correction was applied to *p*-values.

### 2.16 Viral sequence groups and species networks

To investigate microbial association networks between the VSGs that were increased in the antibiotic-exposed fracture patients relative to controls from the DESeq2 analysis and bacterial species, we employed the SparCC algorithm implemented in the *NetCoMi* R package (Peschel et al., 2021) to construct correlation networks separately for individuals with and without antibiotic treatment. Species were filtered at a relative abundance threshold of 0.1 and features with zero variance were removed prior to analysis. SparCC was applied to the 181 retained species (from 3,171) and VSGs to estimate pairwise correlations between them while accounting for the compositional nature of the data. Statistical significance was assessed using 5,000 permutations and resulting *p*-values were Benjamini–Hochberg FDR adjusted. This approach enabled identification of intercorrelations between phages and bacterial species by antibiotic exposure.

### 2.17 Longitudinal analysis of ARGs and phages (rib fracture cohort)

To validate cross-sectional findings and assess temporal links, longitudinal analyses were conducted in a subset of rib fracture patients with paired stool samples collected immediately after antibiotic exposure (hospital discharge; baseline) and again at 6 or 12 weeks (*n* = 10, with five contributing samples at all three timepoints). For each participant, the available post-discharge measurement at 6 or 12 weeks was used as the follow-up measurement; where both 6- and 12-week measurements were available, the mean of the two follow-up measurements was used. ARGs were grouped into on-target (antibiotic-specific) and off-target categories. For descriptive visualization, abundances for each ARG were summed across participants at baseline and follow-up to generate ARG-level trajectories. Separately, patient-level slopes of aggregate on-target and off-target ARG abundances were then estimated using linear regression models and compared between on-target and off-target ARG classes using Welch’s *t*-test. Phage abundances were aggregated per participant and compared between baseline and follow-up using paired *t*-tests. Individual phage trajectories were visualized with spaghetti plots, where each line represented a single participant. To investigate temporal associations between ARGs and VSGs, changes in VSG abundance were calculated (follow-up minus baseline) and correlated with changes in ARG abundance.

### 2.18 Differential enrichment analysis of functional phage and DNA repair gene families

Differential abundance of phage-related and DNA repair-related gene families between fracture cases (frail and non-frail) and controls was assessed using Cliff’s delta, a nonparametric effect size measure, with positive values indicating higher abundance in one group relative to the other and near zero values no difference (Meissel and Yao, 2024). Cliff’s delta was calculated directly from the abundance distributions of each UniRef90 gene family, *p*-values from Wilcoxon rank-sum tests and corrected for multiple testing (Benjamini-Hochberg). Gene families were included only if ≥3 samples were available per group. Results were visualized as bubble plots with Cliff’s delta (x-axis), *p*-value (y-axis), and bubble size proportional to the absolute effect size (Cliff’s delta).

All statistical analyses were performed using R version 4.4.1. Visualizations were prepared using R (RStudio 2024.04.2 + 764), Python v3.13.5, and GraphPad Prism 10.

## 3 Results

The research questions and the basic characteristics of the study cohorts are presented in Figures 1B, 2, respectively. The study included 38 individuals receiving predominantly flucloxacillin- and gentamicin-based prophylactic antibiotics, both targeting aerobic organisms, administered preoperatively to orthopedic trauma patients, as well as 113 antibiotic-free controls. Importantly, we included both healthy and frail controls as individuals living with frailty are known to have different inflammatory responses and microbiome composition than younger healthy individuals.

### 3.1 Antibiotic treatment is associated with increases in individual ARG and VSG abundances

**Figure 3:**
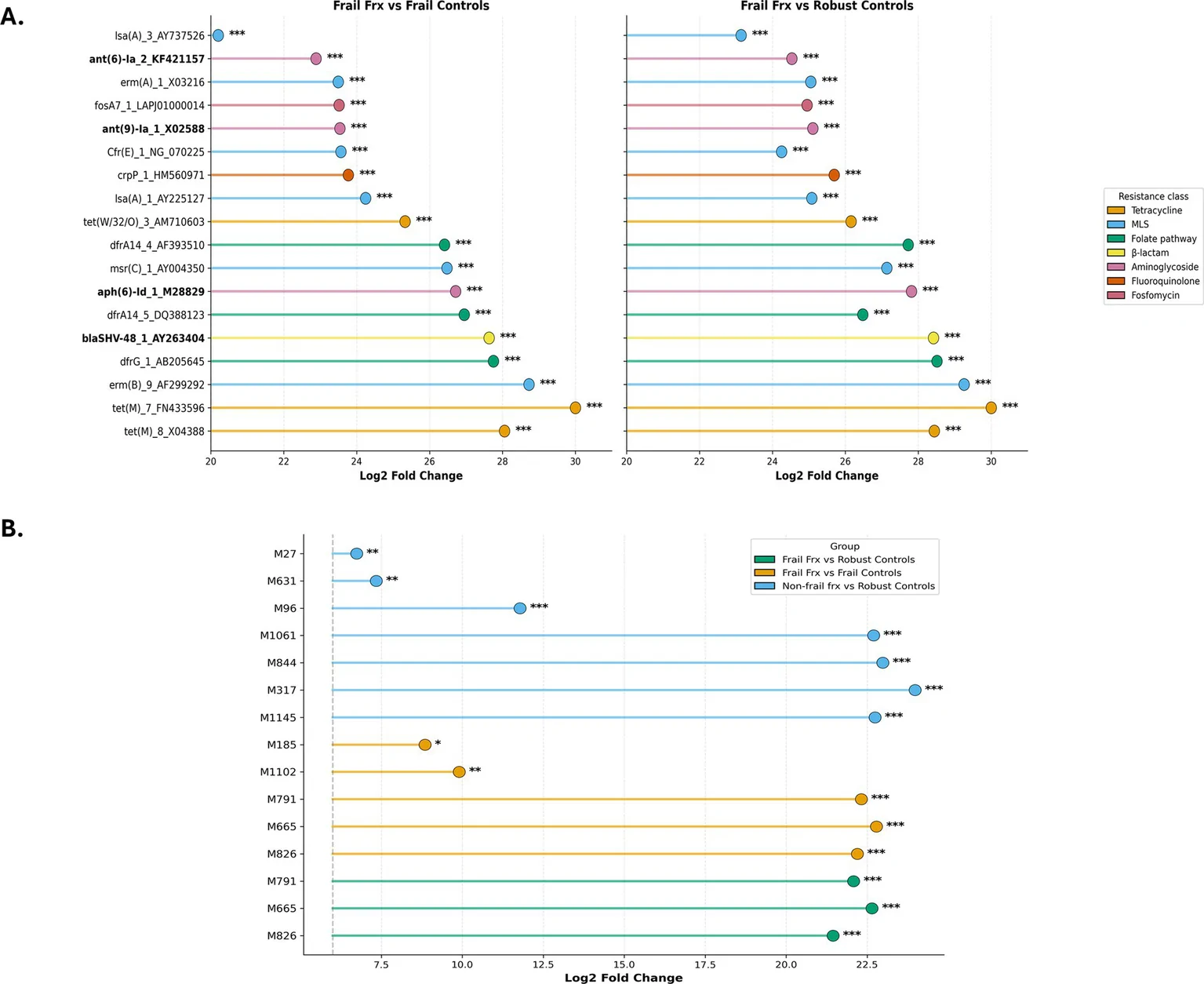
ARG and VSG individual abundances and use of antibiotics. **(A)** Lollipop plot demonstrating ARGs increase in response to preoperative antibiotics in frail fracture orthopedic patients compared to both healthy and frail controls. ARGs are grouped by resistance class. ARGs in bold are “on-target” (conferring resistance to an antibiotic class administered to the corresponding participants) whereas the rest in normal text are off-target (conferring resistance to antibiotic classes not administered). **(B)** Lollipop plot demonstrating VSGs with significantly different abundances in individuals receiving antibiotics vs. controls. Log_2_ fold change (95% CI) and FDR-adjusted *p*-values estimated using DESeq2. M numbers denote VSGs detected across samples. Only VSGs with significantly increased abundance in antibiotic-treated individuals are shown (FDR < 0.05). ARG, antimicrobial resistance genes. VSG, viral sequence group.

Given we previously observed an increase in off-target ARGs (resistance genes for antibiotics not administered) in the “OPERA” cohort (Kouraki et al., 2024), we first assessed whether a similar pattern was present in the frail fracture cohort receiving the same combination of antibiotics. Consistent with our previous results, we observed significant increases in both off-target and on-target (bla, aph, and ant) ARGs in frail fractures compared to frail and healthy controls (Figure 3A). Indeed, we found a larger number of off-target ARGs increasing relative to on-target ARGs in frail hip fracture patients similar to the rib fracture cohort. This was evident in frail hip fracture patients compared to both healthy and frail controls.

To test the hypothesis that increases in phage abundances may contribute to increases in ARGs, we first examined the association between VSG richness and ARG richness. Leveraging samples from antibiotic-treated individuals (*n* = 38) and antibiotic-free controls (*n* = 113), we found a positive association between VSG and ARG richness, adjusting for batch effects, visit, Shannon alpha diversity and confounders (age and sex) (*β* = 0.59 [95%CI 0.026–0.93], *p* = 0.0005, R^2^ = 0.078) (Supplementary Figure S1A). In addition, logistic regression analyses showed that antibiotic exposure was associated with ARG richness (*p* = 0.015), while VSG richness demonstrated a stronger association (*p* < 0.0001), adjusting for batch, visit, Shannon and confounders (Supplementary Figure S1B).

If phages contribute to off-target increases in ARGs, we also expect increases in phages. As anticipated, antibiotic-treated patients showed significantly higher levels of specific VSGs compared to matched controls (Figure 3B) where the M numbers refer to internal identifiers assigned to each VSG in a reference database.

Three individual VSGs (M826, M665 and M791) were >20-fold more abundant in antibiotic-treated frail hip fracture patients, compared to healthy controls. These three increased in the hip fractures patients versus frail controls (*n* = 18) with similarly high-fold changes. In addition, two further VSGs (M1102 and M185) increased in the hip fracture patients versus frail controls with smaller fold changes (9.9 and 8.9, respectively). In antibiotic-exposed rib fracture patients seven VSGs were significantly more abundant compared to healthy antibiotic-free controls from the same batch, with four having >20-fold increases.

### 3.2 Antibiotic treatment is associated with coordinated upregulation of phage and DNA repair response gene families

**Figure 4:**
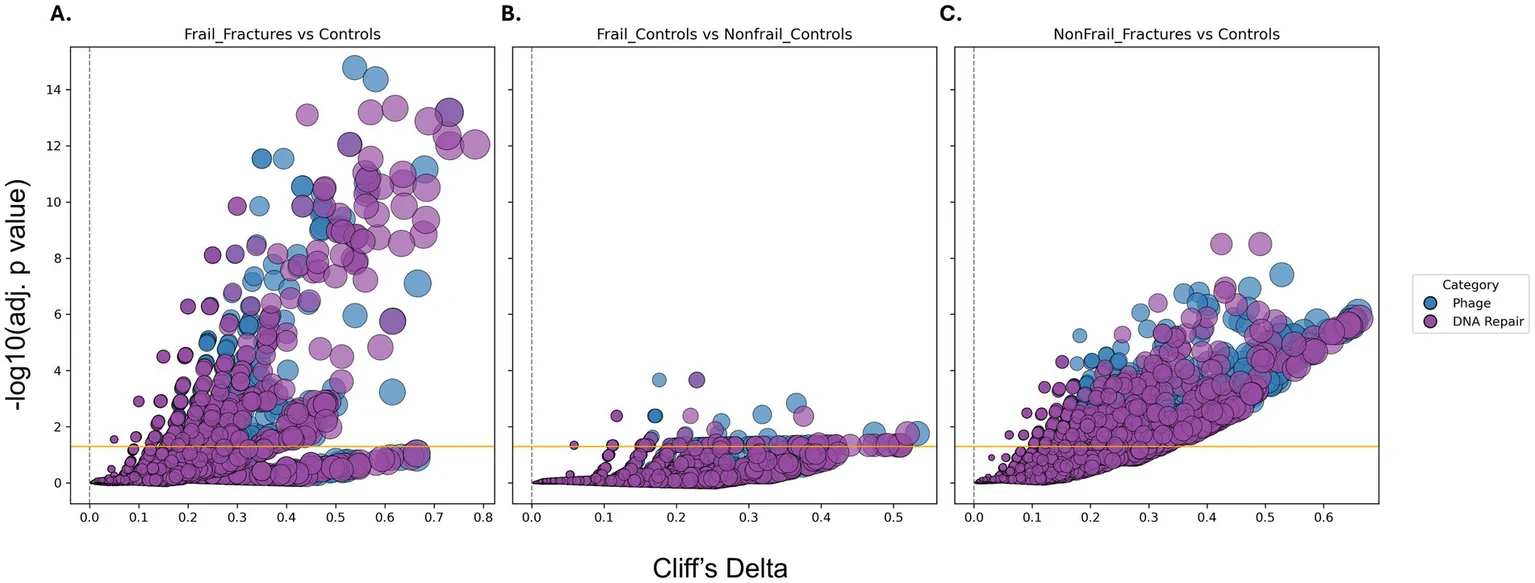
Antibiotic exposure is associated with coordinated upregulation of phage and DNA repair response gene families. Bubble plots showing differential abundance of UniRef90 gene families across comparisons: **(A)** Frail hip fracture patients (antibiotic-exposed) versus controls; **(B)** Frail controls versus healthy controls; **(C)** Non-frail fractures versus controls. Each point represents a gene family, with x-axis showing Cliff’s delta and y-axis showing -log10 (adjusted *p*-value) derived from two-sided Wilcoxon rank-sum tests. Bubble size is proportional to the absolute value of Cliff’s delta (effect size magnitude); colors indicate UniRef90 functional annotation categories: Blue = phage-associated genes, purple = DNA repair pathways. *p*-values were adjusted using the Benjamini–Hochberg false discovery rate (FDR). Horizontal orange lines indicate the significance threshold (FDR < 0.05). Only gene families with at least three samples per group were included in the analysis.

Following our hypothesis, we further expected increases in phage gene families to correspond with increases in DNA repair and SOS response gene families in antibiotic-treated individuals compared to controls. Indeed, comparison of antibiotic-treated patients (frail fractures) versus frail controls showed significant enrichment of phage-associated and DNA damage response pathway gene families (Cliff’s delta > 0.4, FDR *p* < 0.01) (Figure 4A, left panel). A similar pattern was observed in non-frail antibiotic-treated patients compared to healthy controls (Figure 4C, right panel), with hundreds of stress-response and phage-related gene families significantly enriched. Contrarily, frail controls showed minimal differences compared to healthy controls (Figure 4B, middle panel), suggesting that these functional changes are more closely associated with antibiotic exposure than with frailty per se. The coordinated upregulation of phage structural gene families and DNA repair response gene families may support a model wherein antibiotics trigger bacterial SOS responses, contributing to prophage induction and subsequent phage-associated ARG mobilization.

### 3.3 Increases in individual VSGs are linked to increases in off-target ARGs

**Figure 5:**
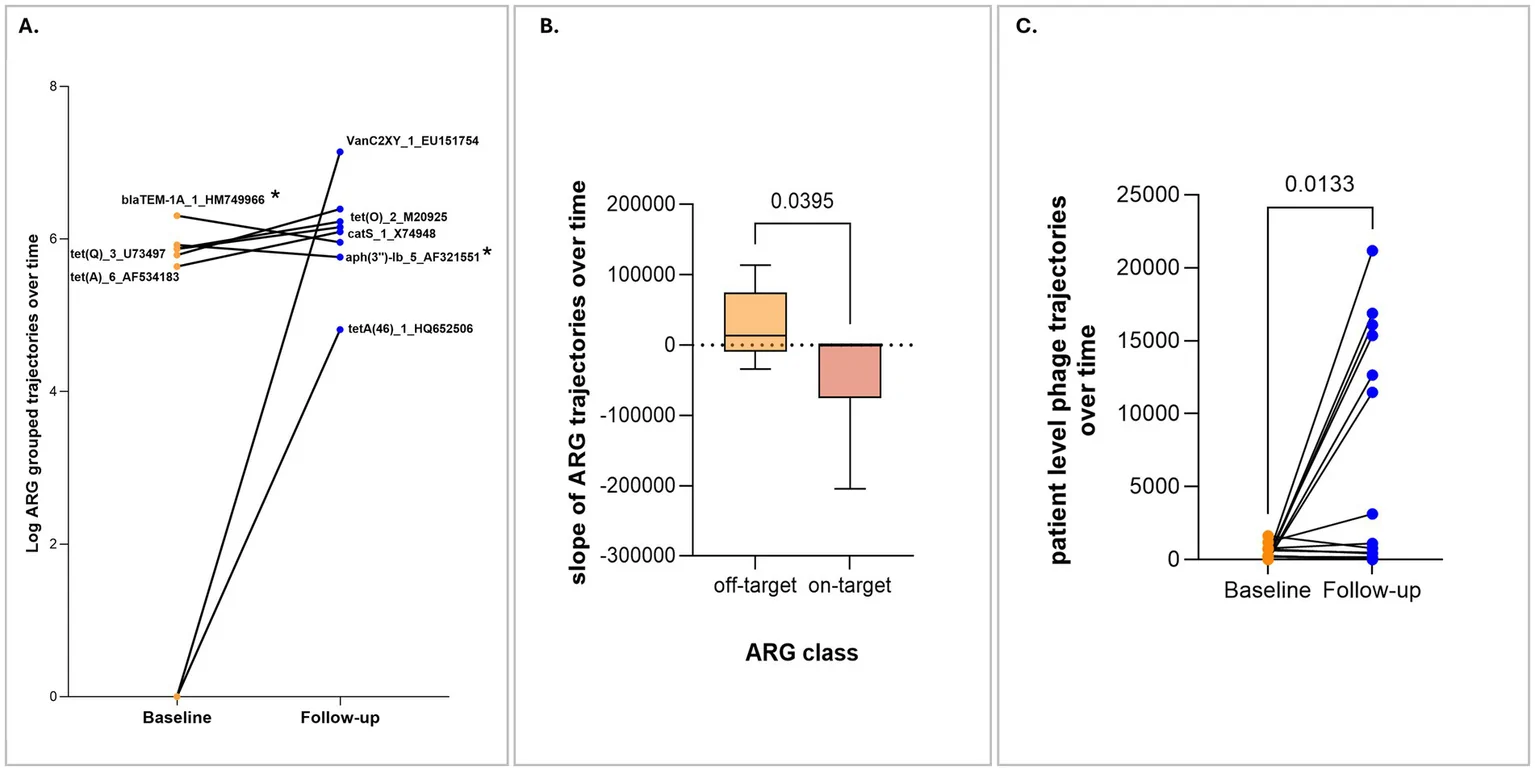
Longitudinal changes in ARGs and VSGs after antibiotic exposure (rib fracture cohort, *n* = 10). **(A)** ARG trajectories from baseline (immediately post-antibiotic) to follow-up at 6 or 12 weeks, grouped by individual ARGs. Off-target ARGs increased over time compared to on-target ARGs (denoted with an asterisk). Each line represents the summed abundance of one ARG across all participants. This panel is descriptive and is intended to illustrate longitudinal ARG trajectories and not a statistical comparison. **(B)** Patient-level slopes of aggregate on-target and off-target ARG trajectories estimated by linear regression, showing a significant difference between off-target and on-target ARG classes. **(C)** Within-participant phage trajectories from baseline to follow-up, assessed by paired comparisons. Each line represents one participant. ARG, Antimicrobial resistance genes; VSG, Viral sequence group.

We next examined longitudinal data available from a subset of antibiotic-treated rib fracture patients (*n* = 10; Figure 2B). Within individuals, off-target ARGs showed consistent increases from discharge to follow-up, while on-target ARGs remained comparatively stable (Figures 5A,B). In parallel, phage abundance also increased between baseline and follow-up in most patients (Figure 5C).

**Figure 6:**
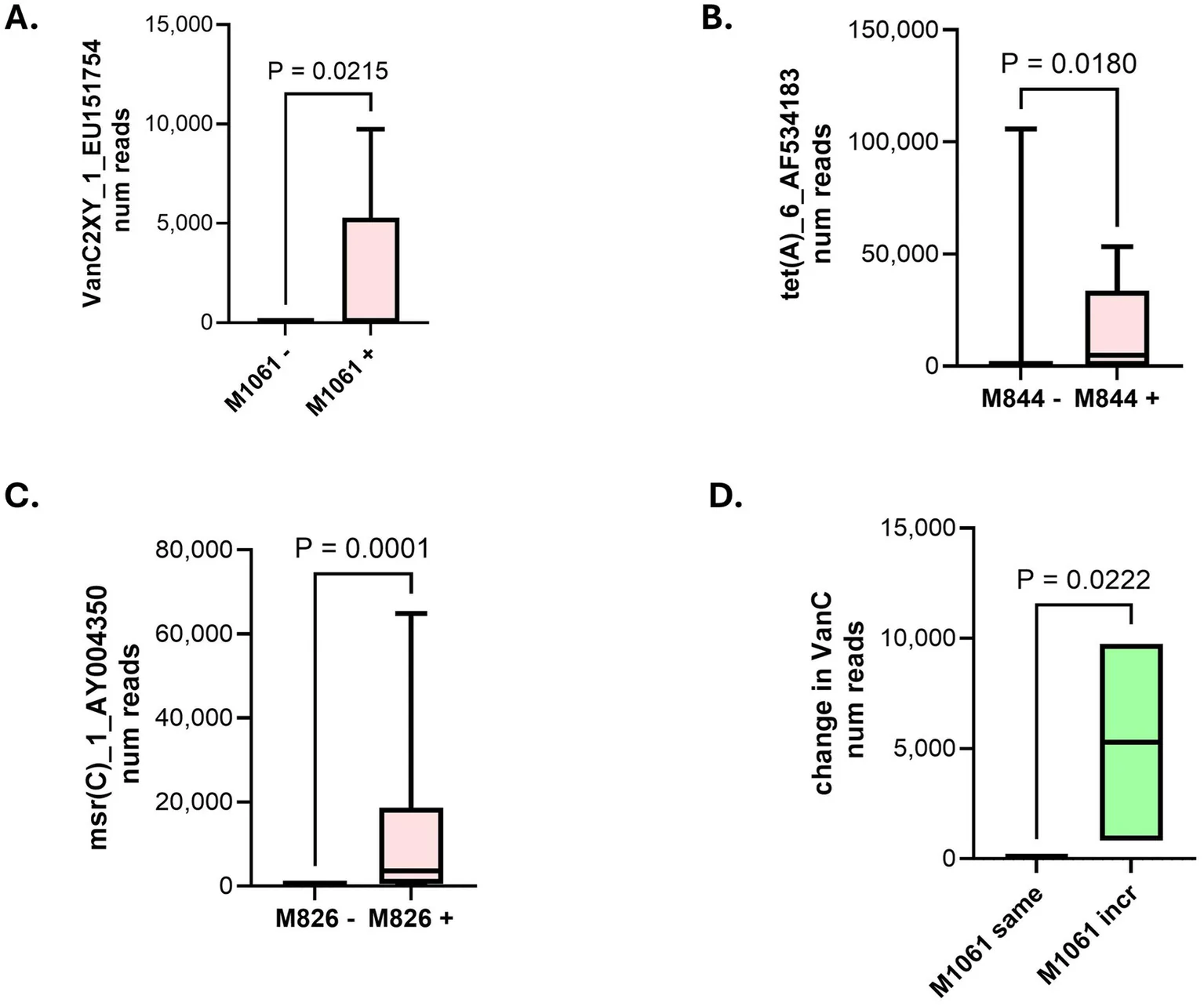
Examples of individual VSGs linked to off-target ARGs. **(A)** Abundance of VanC2XY gene in microbiomes of individuals from the rib fracture cohort carrying or not carrying the M1061 VSG **(B)** Abundance of the tet(A)_6_AF534183 gene patients from the rib fracture cohort carrying or not the M844 VSG **(C)** Abundance of the msr(C)_1_AY004350 in gene patients from the hip fracture cohort carrying or not the M826 VSG **(D)** Increase between time point 1 and time point 2 in the rib fracture cohort of the VanC2XY gene depending on whether there was an increase in the abundance of the M1061 VSG or not during the same period. M numbers denote specific VSGs. ARG, antimicrobial resistance genes; VSG, viral sequence group.

Cross-sectionally, the VSGs which significantly increased in antibiotic-treated patients were also positively correlated with the ARGs that increased in these individuals relative to controls (Supplementary Figure S2A,B). Examples of these are shown in Figure 6. In antibiotic-treated hip fracture patients, elevated VSGs positively correlated with both on-target and off-target ARGs but showed stronger associations with off-target ARGs. We found 10 significant associations with VSGs versus five for on-target ARGs, with higher average correlation strength (Spearman’s Rho = 0.612, 95% CI: 0.326–0.898, vs. 0.533, 95% CI: 0.456–0.610, respectively) (Supplementary Figure S2A).

Longitudinally, increases in VSG abundance were also linked to increases in off-target ARGs (see example in Figure 6D, all the significant correlations are shown in Supplementary Figure S2C). These findings suggest that phage expansion may precede and may be associated with subsequent increases in off-target ARGs after antibiotic treatment. In rib fracture patients, the VSGs elevated after antibiotics exposure were positively correlated with the off-target ARGs, previously shown to increase following antibiotics (Kouraki et al., 2024), and not with any on-target ARGs, supporting an association between specific VSGs and off-target ARGs (Supplementary Figure S2B,C).

### 3.4 Antibiotic treatment links VSG richness to stress and biofilm-related pathways and VSGs to opportunistic species

**Figure 7:**
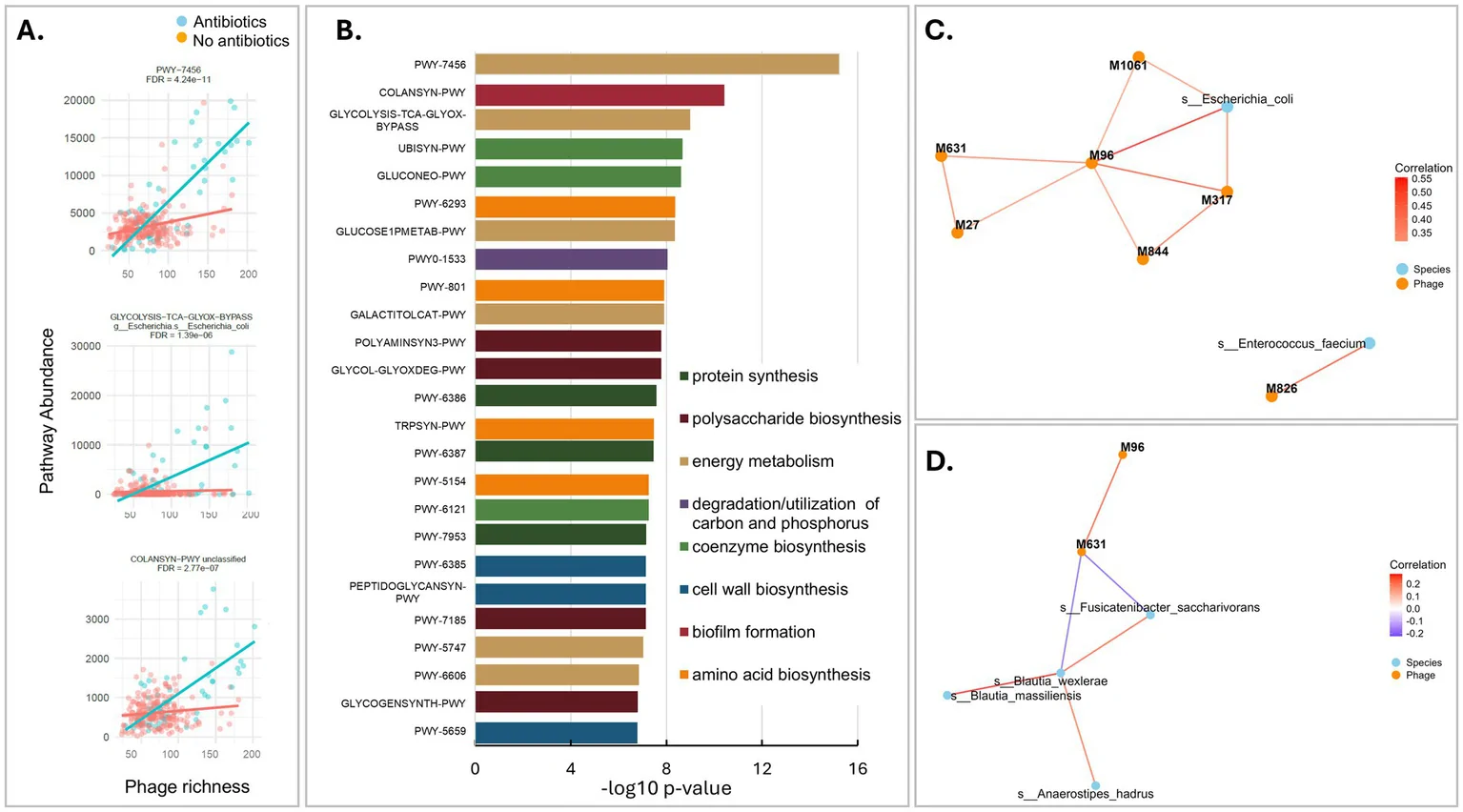
Bacterial pathways and taxa linked to increased phage (VSG) gene richness. **(A)** Scatter plots of correlations between pathway abundance and VSG richness of the three most significant pathways differentially associated with VSG richness in antibiotic-exposed individuals (blue) and antibiotic-free controls (red). **(B)** Bar plot of the top 25 bacterial pathways differentially linked to VSG richness in individuals taking antibiotics compared to controls, with colors indicating functional categories. **(C,D)** Networks of species linked to the significantly increased VSGs from Figure 3 in antibiotic-exposed individuals (top) and antibiotic-free controls (bottom). Only significant SparCC associations (FDR < 0.05, |correlation| > 0.2) are shown. VSG nodes are colored orange, and species are shown in blue. Red edges represent positive correlations, while blue edges indicate negative correlations between VSGs and species. M numbers denote specific VSGs detected across samples.

We further explored whether associations between VSG richness and bacterial pathways differ by antibiotic exposure. A total of 2,160 pathways showed significant interaction effects between VSG richness and antibiotic status (Figures 7A,B). The strongest interaction was observed for PWY-7456: *β*-(1,4)-mannan degradation (FDR *p* = 3.55 × 10^−11^), which was more strongly associated with VSG richness in antibiotic-exposed individuals compared to antibiotic-free controls. Similarly, COLANSYN-PWY: colanic acid biosynthesis (FDR *p* = 2.32 × 10^−7^) showed a stronger positive association under antibiotic conditions, suggesting altered regulation of extracellular polysaccharide production. Finally, the GLYCOLYSIS-TCA-GLYOX-BYPASS pathway driven by *Escherichia coli* (FDR *p* = 1.17 × 10^−6^) similarly showed a stronger positive association under antibiotic conditions.

We next examined whether specific VSGs that increase following antibiotic treatment are associated with bacterial taxa (Figures 7C,D). In antibiotic-treated individuals, VSGs showed numerous and strong correlations both among themselves and with opportunistic species, suggesting tight co-occurrence patterns (Figure 7C). Specifically, VSGs M1061, M96, and M317 were positively correlated with each other and with *E. coli* spp. In addition, M96 and M317 also showed positive correlations with other VSGs, while M826 was only correlated with *Enterococcus faecium* spp. In contrast, the antibiotic-free group showed weaker negative correlations with commensal species (Figure 7D). In this group, VSG M631 was negatively correlated with *Fusicatenibacter saccharivorans* and *Blautia wexlerae* and was positively correlated with VSG M96.

### 3.5 ARG-carrying phages persist months after discharge and are more prevalent in frail than non-frail antibiotic-exposed patients

**Figure 8:**
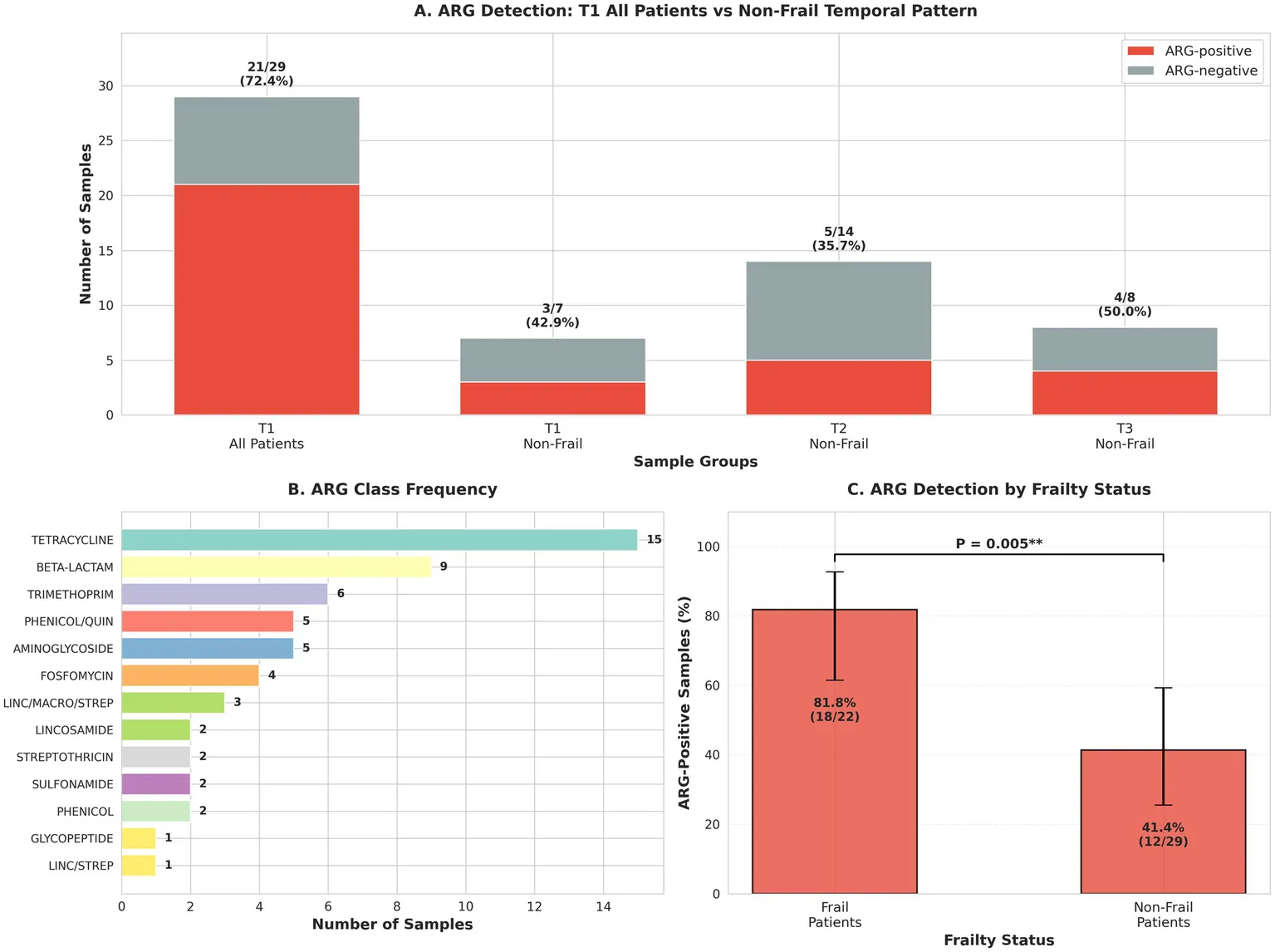
Detection and characterization of antimicrobial resistance genes in viral contigs from antibiotic-exposed trauma patients. **(A)** Sample-level ARG detection by timepoint. Stacked bars show the number of samples with (red) and without (gray) ARG-carrying viral contigs at discharge for both cohorts (frail and non-frail) and ARG-carrying viral contigs only for the non-frail fracture cohort at discharge (T1), 6 weeks (T2), and 12 weeks (T3) post-surgery. Labels indicate number of ARG-positive samples, total samples, and percentage. **(B)** ARG class frequency across samples. Horizontal bar chart showing the number of samples containing each ARG class. Linc/macro/strep, lincosamide/macrolide/streptogramin; phenicol/quin, phenicol/quinolone; macro/strep, macrolide/streptogramin; linc/strep, lincosamide/streptogramin. **(C)** ARG detection by frailty status with confidence intervals. Bar plot showing percentage of ARG-positive samples in frail versus non-frail trauma patients. *p*-value from Fisher’s exact test. ARG, antimicrobial resistance genes.

Finally, to examine whether phages may harbor off-target ARGs, we performed assembly-based viromics analysis on 51 samples from 38 antibiotic-exposed trauma patients. ARG-carrying viral contigs were detected in 30 of 51 samples (58.8%), with 57 viral sequences harboring 78 resistance genes across 13 resistance classes (Figure 8).

ARG carriage did not change over time and similar detection rates of ARGs in phages were seen at T1 (discharge) as well as at 6 and 12 weeks post-discharge (T1: 42.9%, T2: 35.7%, T3: 50.0%, *p* > 0.05.) in the non-frail fracture cohort where longitudinal data was available (Figure 8A). These data suggest that ARG-carrying phages may persist in the guts of a substantial proportion of patients months after discharge.

The predominant classes were tetracycline (15 samples), beta-lactam (9 samples), and trimethoprim (6 samples). ARGs showed 92–100% sequence identity to reference genes, and some contigs carried multiple resistance determinants (mean 1.37 genes/contig), including complete resistance operons (Figure 8B). Overall, 13.3% (4/30) of contig-carrying ARGs carried only on-target (beta-lactam or aminoglycoside) ARGs, 30% (9/30) carried both on- and off-target ARGs and 56.7% (17/30) carried only off-target ARGs.

We also find that ARG-carrying viral contigs were significantly more prevalent in frail patients (81.8%, 18/22) compared to non-frail patients (41.4%, 12/29; *p* = 0.005) (Figure 8C).

This additional genomic evidence, combined with our previous analyses showing increased individual VSG abundances as well as increased VSG richness post-antibiotics, supports the presence of ARGs on viral contigs and is consistent with a potential role for phages in ARG expansion in antibiotic-exposed patients.

## 4 Discussion

In this study, we show that following perioperative antibiotics, increases in ARGs were not limited to on-target resistance but were also observed for a broad range of off-target ARGs, which were linked to increases in phage abundances (VSGs). Consistent with our previous findings in antibiotic-treated rib fracture patients (Kouraki et al., 2024), frail fracture patients receiving predominantly flucloxacillin- and gentamicin-based prophylactic antibiotics showed a more pronounced increase in off-target than on-target ARGs compared to both healthy and frail controls.

Our results are particularly noteworthy in light of recent work suggesting that antibiotics targeting aerobic organisms may pose a lower risk of off-target resistance expansion (Heston et al., 2024). Despite the use of predominantly aerobic-targeting antibiotics in our cohorts, we observed substantial increases in off-target ARGs, suggesting that the risk of resistance expansion may extend beyond the aerobic/anaerobic spectrum. One possible explanation is that bacterial stress responses and prophage activation, rather than direct target spectrum alone, may contribute to ARG expansion following antibiotic administration. While previous studies have demonstrated that gut phages can carry ARGs in healthy populations (Quirós et al., 2014; Brown-Jaque et al., 2018), data linking phages and ARGs in clinical cohorts exposed to prophylactic antibiotics remain limited.

Critically, we observed that VSG gene richness showed a stronger association with antibiotic exposure than ARG richness, and specific VSGs correlated more strongly with off-target than on-target ARGs. These findings, validated in a subset of patients with longitudinal data, are consistent with a model where bacterial stress following antibiotic treatment may promote phage activation and a potential role for phages in horizontal transfer of resistance genes (Borodovich et al., 2026; Münch et al., 2023; Modi et al., 2013; Maurice et al., 2013).

An alternative, non-mutually exclusive explanation for the observed off-target ARG expansion is selection of bacterial strains that are inherently resistant, rather than phage-mediated horizontal transfer. Shotgun metagenomic profiling alone cannot definitively distinguish these mechanisms, as it does not directly localize a resistance gene to a chromosomal, plasmid, or phage context. However, several observations in our data are more consistent with a phage-associated contribution than with strain selection alone: ARGs were directly detected on quality-filtered, assembled viral contigs using an independent pipeline, providing physical evidence of phage-encoded resistance genes; increases in VSG abundance preceded or coincided with subsequent increases in off-target ARG abundance within individuals over time; phage-associated and DNA-repair/SOS-response gene families were coordinately enriched following antibiotic exposure, consistent with prophage induction; and off-target ARGs were detected across a broad range of bacterial taxa rather than restricted to a single expanding clone. We cannot, however, formally exclude a contribution from strain-level selection, and strain-resolved metagenomics, phage-host linkage approaches (e.g., CRISPR spacer matching or proximity-ligation sequencing), and culture-based validation represent important next steps to directly test phage-mediated transfer versus selection of resistant organisms.

These findings extend observations from traveler studies, where antibiotic use increases acquisition of multidrug-resistant Enterobacterales through microbiome disruption and selection of resistant strains (DuPont and Steffen, 2017; Ruppé et al., 2018; Furuya-Kanamori et al., 2020). Here, we demonstrate a mechanistic correlate of this phenomenon in a clinical perioperative context, supporting the idea that even short courses of prophylactic antibiotics could potentially create ecological conditions that favor the emergence and persistence of resistance, not only through resistant bacteria as observed in travelers, but also by phage-associated dissemination of ARGs within the gut resistome.

At the functional level, antibiotic exposure modified the relationship between phage diversity and microbial metabolic pathways. Specifically, three pathways, *β*-(1,4)-mannan degradation (PWY7456), COLANSYN-PWY, and the GLYCOLYSIS-TCA-GLYOX-BYPASS superpathway, involved in nutrient metabolism, stress response and biofilm formation, and energy production, respectively, were significantly associated with VSG gene richness in antibiotic-treated individuals. The positive association with *β*-(1,4)-mannan degradation may reflect phage-mediated lysis of mannan-degrading taxa (Dawood and Ma, 2020; Fernandes and São-José, 2018). Concurrently, the GLYCOLYSIS-TCA-GLYOX-BYPASS superpathway, enriched in *Escherichia coli*, may suggest phage manipulation of host metabolism to facilitate replication or an adaptive host response to phage and antibiotic stress (Li et al., 2024). The COLANSYN-PWY pathway, which governs colanic acid biosynthesis (a key component of the extracellular matrix in Enterobacteriaceae) also displayed a strong interaction with VSG gene richness under antibiotic conditions. Given colanic acid’s upregulation following antibiotic exposure (Piché et al., 2025) and its role in mediating biofilm formation (Wang et al., 2020), its upregulation could potentially indicate phage exploitation of biofilm-mediated survival strategies. The above suggest that phage activity may interact with metabolically active, antibiotic-resistant taxa, potentially linking phage expansion to functional shifts in the microbiome under antibiotic pressure.

These functional shifts were mirrored at the species level. In antibiotic-treated individuals, VSGs showed more frequent positive correlations with opportunistic species such as *E. coli* and *Enterococcus faecium*, consistent with the enrichment of central metabolic and stress-related pathways characteristic of these taxa. VSGs M1061, M96, and M317 were not only positively correlated with each other but also with *E. coli*, suggesting the possibility of linked phage-host associations that may reflect targeted predation or prophage induction under antibiotic stress (Buttimer et al., 2022). In contrast, correlations in antibiotic-free individuals were weaker and only with commensals (i.e., *Fusicatenibacter saccharivorans* and *Blautia wexlerae*). Taken together, these patterns suggest that antibiotic treatment may amplify the ecological influence of phages, enabling them to modulate both microbial structure and function, particularly under conditions of antibiotic stress (Kurilovich and Geva-Zatorsky, 2025).

Consistently, both phage-associated genes and DNA damage response genes were significantly enriched in antibiotic-treated individuals, compared to controls, including both healthy and frail controls [with frail expected to have a higher systemic inflammatory status (Soysal et al., 2016)].

To provide further validation, we applied an independent assembly-based viromics pipeline, which identified ARGs on viral contigs in 60% of antibiotic-exposed patients. ARG carriage was nearly 2-fold higher in frail patients, likely reflecting frailty’s multidimensional pathophysiology including chronic inflammation, multimorbidity, dysbiotic microbiome, and prior antibiotic exposure (Soysal et al., 2016; Clegg et al., 2013; Claesson et al., 2012). These factors may enhance prophage induction and ARG maintenance, suggesting that prophages may act as reservoirs of resistance genes especially in vulnerable patient populations.

We hypothesize that this pattern may reflect two additive mechanisms rather than a single selective process. Mobile genetic elements frequently carry multiple resistance genes as cassettes. Direct antibiotic selection of on-target ARG-carrying elements would therefore be expected to co-amplify off-target ARGs residing on the same elements to a comparable degree. However, antibiotic-induced bacterial stress additionally triggers induction of lysogenic phages independent of their ARG content, providing a second, cassette-independent route of amplification for elements carrying only off-target ARGs. This combined effect of co-selection stress-induced lysogenic release offers a potential explanation for the stronger off-target association. It is also consistent with the coordinated enrichment of phage-associated and DNA-repair/SOS-response gene families observed specifically in antibiotic-exposed individuals (Figures 4A,C) but not between frail and healthy controls (Figure 4B), suggestive of an antibiotic-specific rather than frailty-driven induction effect.

We note several study strengths. First, the controlled nature of the design with individuals receiving the same combination of prophylactic antibiotics. Second, the use of deep sequencing metagenomic data allowing us to link taxonomy, function, ARGs and VSGs and the availability of age/frailty-matched controls adding robustness to our design. Third, longitudinal sampling in a subset of antibiotic-exposed fracture patients, the direct identification of ARGs on assembled viral contigs with an independent pipeline and functional enrichment analyses, which further strengthens this study.

We also note some study limitations. Our cohorts, and in particular the longitudinal subset of rib fracture patients (*n* = 10, with paired baseline and follow-up data available for five participants across all three timepoints), are relatively small. This limits statistical power, increases the likelihood that modest or heterogeneous effects were missed, and means that individual correlation and slope estimates should be interpreted with caution given their wide confidence intervals. Accordingly, the longitudinal findings presented here should be regarded as hypothesis-generating. Larger, adequately powered and, ideally, independent validation cohorts are needed to confirm the magnitude and consistency of the phage-ARG associations we describe, and to determine whether the trajectories observed in this subset generalize to the wider antibiotic-exposed trauma population. The absence of pre-antibiotic baseline samples limits causal inference, and direct functional validation of phage-mediated ARG transfer was not performed. Nevertheless, our findings are consistent with previously described roles of gut phages in horizontal gene transfer (Borodovich et al., 2022). All participants were recruited from a single UK Level 1 Trauma Centre (Nottingham University Hospitals NHS Trust) and antibiotic-treated patients received a broadly consistent perioperative regimen (predominantly flucloxacillin and gentamicin). While this homogeneity strengthens internal validity by limiting confounding from heterogeneous antibiotic exposures, it limits the generalisability of our findings to other institutions, patient populations, and prophylactic regimens. Local antibiotic stewardship policies, case-mix, and baseline gut microbiome composition can vary substantially between centers and healthcare systems, and other commonly used perioperative antibiotics (e.g., cephalosporins or co-amoxiclav) may have different effects on phage-ARG dynamics than those observed here. Multi-centre studies encompassing a broader range of antibiotic regimens, patient demographics, and geographic settings are needed to establish the generalisability of these findings before they can inform antibiotic stewardship policy more broadly. The low expected incidence of surgical site infections (<2%) in closed fracture cases, prevented direct associations with clinical outcomes. Accordingly, our analyses were designed to generate mechanistic insights into antibiotic-associated phage and ARG dynamics. Moreover, VSGs were derived from metagenomic sequencing data rather than from dedicated virome-enriched sequencing, which preferentially captures prophages integrated within bacterial genomes rather than free viral particles (Li et al., 2025). Nevertheless, the observed enrichment of phage and DNA damage response-associated genes is consistent with prophage activation following antibiotic exposure. Future studies incorporating VLP-enriched viromes would provide complementary insight into extracellular viral dynamics (Li et al., 2022). In addition, the VSGs discussed here are identified by internal MetaPhlAn reference-database identifiers rather than an established viral taxonomy, and the large majority (85.1% of the full VSG catalogue) lack a match to a characterized RefSeq viral genome (Zolfo et al., 2024). We therefore cannot state with certainty whether the specific ARG-associated VSGs highlighted in this study (e.g., M1061, M96, M317, M826) correspond to previously described phage species as none of them had a characterized RefSeq viral genome match. Their putative bacterial hosts (*E. coli* for M1061, M96 and M317; *Enterococcus faecium* for M826) were inferred indirectly from co-occurrence network analysis (Figures 7C,D) rather than from sequence-based host-prediction methods, and should therefore be regarded as statistical associations rather than confirmed host assignments. Dedicated host-prediction approaches, such as CRISPR spacer matching or oligonucleotide-composition-based tools, alongside culture-based phage isolation, would help confirm the bacterial hosts of these ARG-linked viral sequence groups in future work. While inflammation in trauma patients may contribute to some effects, our previous work showed increased off-target ARGs not only versus healthy controls but also versus trauma patients without antibiotics (Kouraki et al., 2024). Consistently, functional analyses revealed no difference between frail (high inflammation) and healthy controls, suggesting that the observed effects are more likely to be associated with antibiotic exposure. Finally, our metagenomic approach does not quantify absolute VSG or ARG abundances and all abundance-based analyses are relative and compositional. Although we employed richness metrics and metagenomic-specific analytical frameworks to reduce compositional bias, these approaches remain dependent on sequencing depth and detection thresholds.

## 5 Conclusion

Despite these limitations, this is, to the best of our knowledge, the first study to identify links between phages and ARGs as well as links to specific microbial pathways in patient populations. The observed phage-ARG associations provide mechanistic insight into how prophylactic antibiotics may reshape the gut resistome and have important implications for perioperative antibiotic use. The preferential expansion of off-target ARGs suggests that prophylactic antibiotics may inadvertently promote resistance to antibiotics not administered, potentially impacting future treatment options.

In conclusion, our findings are consistent with a model in which antibiotic exposure is associated with bacterial stress responses and prophage activation, potentially increasing phage abundance. This process may contribute to the dissemination of off-target ARGs. Importantly, our results show that even antibiotics directed at aerobes, which have been hypothesized to carry a lower risk of off-target resistance expansion, were associated with substantial increases in VSGs and off-target ARGs. Further functional studies are needed to confirm the mechanistic role of phages in ARG dissemination following antibiotic treatment and to determine the clinical significance of these resistance changes.

## Data Availability

The datasets presented in this study can be found in online repositories. The names of the repository/repositories and accession number(s) can be found at: https://www.ncbi.nlm.nih.gov/, PRJNA1110178.
