# Supplementary material for: Gut phages are linked to off-target antimicrobial resistance genes in orthopedic patients receiving peri-surgical antibiotics
Source: Front Microbiol. 2026 Sep 9;17:1892653. doi: 10.3389/fmicb.2026.1892653 (PMC13597856; doi:10.3389/fmicb.2026.1892653)
Supplement: Supplementary file 1 [file Table_1.docx]

Supplementary Material

# 1 Supplementary Figures and Tables

## 1.1 Supplementary Tables

**Table S1.** Inclusion and exclusion criteria for the Link between Frailty and Gut Microbiome in Older Adults (FLORA) study

**Inclusion criteria**

- Participants who have responded to IMH&W with a complete FRAIL scale
- Age ≥ 60 years.

**Exclusion criteria**

- Insufficient understanding of spoken or written English to meet the requirements of the study protocol
- Inability to meet the requirements of research assessments
- Major active psychiatric condition, e.g. major depression
- Unable to give informed consent
- Unstable angina or severe heart failure (class ≥3)
- Acute medical illness (for example, recent major operation or stroke, or any hospital admission) within three months

**Table S2.** Details of specific antibiotics and duration administered to surgical patients admitted to hospital for multiple rib fractures and hip fractures versus controls

|  | n | Antibiotics ^(1)^ % | | | |
| --- | --- | --- | --- | --- | --- |
|  |  | *Flucloxacillin 24hrs* | *Gentamicin*  *1 dose* | *Tazocin 1dose* | *Co-amoxiclav 5doses* |
| Controls | 113 | 0.0% | 0.0% | 0.0% | 0.0% |
| Fracture patients antibiotics | 38 | 100.0% | 97.4% | 7.9% | 2.6% |

(1) Flucloxacillin is a narrow-spectrum beta-lactam antibiotic belonging to the penicillin class. It is commonly used to treat infections caused by susceptible Gram-positive bacteria, including *Staphylococcus aureus.* Gentamicin is an aminoglycoside antibiotic that acts by inhibiting protein synthesis in susceptible bacteria. It is effective against a wide range of Gram-negative bacteria and some Gram-positive bacteria. Tazocin, also known as piperacillin/tazobactam, is a combination antibiotic containing piperacillin, a broad-spectrum penicillin, and tazobactam, a beta-lactamase inhibitor. It is used to treat a variety of infections caused by susceptible bacteria, including both Gram-positive and Gram-negative organisms. Co-amoxiclav, also known as amoxicillin/clavulanic acid, is a combination antibiotic consisting of amoxicillin, a penicillin-like antibiotic, and clavulanic acid, a beta-lactamase inhibitor. It is effective against a wide range of bacterial infections, including those caused by beta-lactamase-producing bacteria, and is commonly used for respiratory tract infections, urinary tract infections, and skin infections.

## 1.2 Supplementary Figures

**Figure S1**. (A) Scatter plot of the association between gene richness for ARGs and VSGs (B) Forest plot of odds ratios (95% C.I.) reflecting per-unit changes in the predictor variables from logistic regression testing the association between VSG gene richness, ARG richness and use of antibiotics

**Figure S2**. **Individual VSGs are linked to ARGs**. (A–B) Heatmaps of correlations between on- and off-target ARGs and VSGs that increased after antibiotic exposure in (A) frail hip fracture individuals and (B) non-frail rib fracture individuals. (C) Heatmap of changes from baseline (immediately post-antibiotic exposure) to follow-up in non-frail rib fracture individuals, showing that increases in VSG abundance paralleled changes in ARGs, supporting a temporal link.  M numbers denote Viral Sequence Groups (VSGs) detected across samples. * p<0.05 ** p<0.01 *** p<0.001
